# Generalized additive mixed models for disentangling long-term trends, local anomalies, and seasonality in fruit tree phenology

**DOI:** 10.1002/ece3.707

**Published:** 2013-08-02

**Authors:** Leo Polansky, Martha M Robbins

**Affiliations:** Max Planck Institute for Evolutionary AnthropologyDeutscher Platz 6, 04103, Leipzig, Germany

**Keywords:** Bwindi Impenetrable National Park, climate change, nonparametric models, phenology, plant reproductive ecology, tropical forest ecology

## Abstract

Quantifying temporal patterns of ephemeral plant structures such as leaves, flowers, and fruits gives insight into both plant and animal ecology. Different scales of temporal changes in fruits, for example within- versus across-year variability, are driven by different processes, but are not always easy to disentangle. We apply generalized additive mixed models (GAMMs) to study a long-term fruit presence–absence data set of individual trees collected from a high-altitude Afromontane tropical rain forest site within Bwindi Impenetrable National Park (BINP), Uganda. Our primary aim was to highlight and evaluate GAMM methodology, and quantify both intra- and interannual changes in fruit production. First, we conduct several simulation experiments to study the practical utility of model selection and smooth term estimation relevant for disentangling intra- and interannual variability. These simulations indicate that estimation of nonlinearity and seasonality is generally accurately identified using asymptotic theory. Applied to the empirical data set, we found that the forest-level fruiting variability arises from both regular seasonality and significant interannual variability, with the years 2009–2010 in particular showing a significant increase in the presence of fruits-driven by increased productivity of most species, and a regular annual peak associated occurring at the end of one of the two dry seasons. Our analyses illustrate a statistical framework for disentangling short-term increases/decreases in fruiting effort while pinpointing specific times in which fruiting is atypical, providing a first step for assessing the impacts of regular and irregular (e.g., climate change) abiotic covariates on fruiting phenology. Some consequences of the rich diversity of fruiting patterns observed here for the population biology of frugivores in BINP are also discussed.

## Introduction

How ephemeral plant structures such as leaf, flower, and fruit presence vary through time and space are important for many branches of ecology and sociobiology. These patterns provide clues to determinants and strategies of plant reproductive ecology (Rathcke and Lacey 1985) as well as motivate theoretical studies about consumer population trajectories and genotype evolution in variable environments (Levins 1969; Boyce and Daley 1980; Holt 2008). Eruptions in fruit abundance such as those associated with, but not limited to, masting can have cascading impacts on populations and behavior across trophic levels (Schmidt and Ostfeld 2008) and they can act as ecological constraints that may shape the evolution of social structure and cooperative living (Hatchell and Komdeur 2000). Thus, scientists are often led to ask the following question: How is fruit production changing on different temporal scales? This study examines approaches to quantitatively detect and disentangle long-term and interannual differences from regular seasonal variation in ephemeral plant structures.

The mechanisms leading to patterns of fruit production at the species and forest community level are diverse. These include intraannual seasonal periodicity in abiotic climatic variables, such as light, rainfall, and temperature (Rathcke and Lacey 1985; Wright and van Schaik 1994; Reich 1995; Lewis et al. 2004); relatively low-frequency environmental perturbations related to, for example, El Niño events (Wright and Calderón 2006); internal resource allocation strategies (Isagi et al. 1997; Satake and Iwasa 2002); and biotic economies of scale such as pollination efficiency or predator satiation (Silvertown 1980; Ims 1990; Kelly and Sork 2002).

Methods to quantify fruit production are diverse and have utilized a wide range of strategies. Historically, methods have included the use of information theory (Colwell 1974); classical summary statistics such as the coefficient of variation (Kelly and Sork 2002); time-series tools using both regression on trigonometric functions (i.e., Fourier type analysis—see Chapman et al. 1999 and Norden et al. 2007) and lagged values of the observations themselves (i.e., examination of autocorrelation functions—see Koenig and Knops 2002); and graphical observations (Chapman et al. 2005). These methods have relative strengths that depend on the context of the system and question (Crone et al. 2011), but explicitly describing both seasonal and trend components, and conducting inferential hypothesis testing to identify parsimonious models is difficult, if possible at all, in many of these approaches. For example, classical summary statistics do not address the temporal dependencies at all, whereas Fourier analysis which does provide a natural approach to modeling periodic phenomena (Shumway and Stoffer 2000) does not allow the potential for a random-effects model structure (e.g., needed when seed traps or individual specimens are repeatedly surveyed) or an easy evaluation of the significance of covariates sensu classic regression analyses.

Given the diverse patterns of fruit cycles and abundance observed in empirical data (e.g., Sakai 2001; Chapman et al. 2005; Polansky and Boesch 2013), models explaining them in single- and multiple-species analyses should have some flexibility (Hudson 2010; Polansky and Boesch 2013). A recent text edited by Hudson and Keatley (2010) synthesizes many of the modern statistical approaches relevant for phenology studies. Of these approaches, nonparametric smoothing has been proposed as a way to link predictor variables with phenology data to identify nonlinearity in phenological responses without a priori specification of the exact structure (Hudson 2010; Hudson et al. 2010, 2011; Roberts 2010); nonparametric smoothing has also been identified as particularly useful technique for controlling for seasonal fluctuations in tropical phenology data sets in an attempt to isolate long-term linear trends (Polansky and Boesch 2013). Here, we focus on the utility and applications of both generalized additive models (GAMs) and generalized additive mixed models (GAMMs) for phenological data analysis. These frameworks are flexible and easy to implement, but have received relatively little attention in phenological studies (but see Gaira et al. 2011 for a study of flowering times using GAMs). In particular, it is particularly easy to implement models capturing the periodic properties of phenology using GAMs (via the use of a cyclic basis) which is not always the case with other nonparametric techniques.

With an eye toward practical application, we first consider two shortcomings particularly relevant to phenological data that might strain the approximations used in statistical inference in smoothing approaches. First, sampling interval designs for long-term studies is often too coarse to identify smooth changes. For example, while monthly sampling schemes are often the realistic limit of sampling effort over multiyear timescales, it cannot be expected to provide perfect information about events that happen over the course of days or several weeks such as rapid and complete emergence of flowers or fruits. This means that the target function for which the GAM approach is asked to estimate is not smooth on the scale of the data in hand. Second, overall sample sizes may be relatively small even for long multiyear studies, in contrast with sample sizes in simulation studies typically used to test how equations derived using large sample theory perform in practice.

We then apply GAMMs to study nearly 8 years of monthly collected fruiting presence–absence data from polycarpic plants of a high-altitude Afromontane rainforest located in Bwindi Impenetrable National Park (BINP), Uganda. A random-effects (mixed) model structure is important here because individual plants are repeatedly surveyed. Data points are dependent not only on time but also on the individual surveyed because tree-level factors such as local soil, shade, disturbance, or age can potentially impact individual reproductive effort. Here, our primary goals are to quantitatively identify years with significantly higher fruit production, test for the significance of seasonality given regular seasonal variation in rainfall, and to test for evidence of long-term increases in the proportion of individuals with fruits as found in a nearby lowland tropical forest within Kibale National Park by Chapman et al. (2005).

## Material and Methods

### Nonparametric models

We first provide a brief review of the theory underlying the techniques used here, which may be skipped. Readers interested in the theory of GAMs are recommended to the text by Wood (2006) and citations therein. A GAM (Hastie and Tibshirani 1986) extends the generalized linear model (GLM) by allowing the predictor function to also include a priori unspecified nonlinear functions of some or all the covariates. Given *n* observations of a random variable *y*_*i*_, *i* = 1, …, *n*, from a member of the exponential family of distributions and a link function *g*, the basic deterministic structure of a GAM is


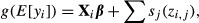
(1)

where **X**_*i*_***β*** is the linear parametric component of the model with **X**_*i*_*,* the *i*th row of the design matrix **X** associated with covariates that are modeled linearly to *y*_*i*_ as in a GLM, and *s*_*j*_(*z*_*i,j*_) are smooth and nonlinear functions of predictor variables ***z***_*j*_. If no linear component is included, then the model is referred to as nonparametric, whereas a model whose predictions consist of both linear and unspecified nonlinear functions of predictor variables is often referred to as a semiparametric model.

One decomposition of the right-hand side of equation 1 relevant to phenological data is into two disparate components, one to capture long-term cumulative changes such as might be associated with large-scale climate anomalies, and a second related to shorter term drivers such as those patterns associated with regular seasonal variation in rainfall, temperature, or irradiance. Incorporating these notions into equation 1 produces the model


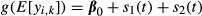
(2)

where ***β***_0_ is the intercept, *s*_1_(*t*) is a smooth function of the time *t* since the start of the study, *s*_2_(*t*) is a cyclic function. More parsimonious models contained within equation 2 that are studied here are summarized in Table 1, which are produced by singularly removing the model terms. We also include a semiparametric model (Table 1, Model 2) which constrains the interannual smooth *s*_1_(*t*) to be linear, such as might describe long-term persistent and directional changes in the expected response *E*[*y*_*i,k*_].

**Table 1:** Summary of the core model structures

Models	Equation	Description
Model 1 (M1)	*g*(E[*y*_*i,k*_]) ∼ *β*_0_ + *s*_1_(*t*) + *s*_2_(*t*)	Nonlinear trend and seasonal production
Model 2 (M2)	*g*(E[*y*_*i,k*_]) ∼ *β*_0_ + *β*_1_*t* + *s*_2_(*t*)	Linear trend and seasonal production
Model 3 (M3)	*g*(E[*y*_*i,k*_]) ∼ *β*_0_ + *s*_1_(*t*)	Nonlinear trend only
Model 4 (M4)	*g*(E[*y*_*i,k*_]) ∼ *β*_0_ + *s*_2_(*t*)	Seasonal production (no significant trend)
Model 5 (M5)	*g*(E[*y*_*i,k*_]) ∼ *β*_0_	Null random-effects model

The empirical data models also included temporal autocorrelation and random-effects terms.

There are several options for selecting a basis, a collection of functions which can be added together to estimate *s*_*j*_, for each of the *s*_*j*_ (see Ch. 3–4 and p. 212–217 in particular in Wood 2006 for a summary). Experience and the need to require *s*_2_ to be cyclic suggest using cubic splines as basis functions for each *s*_*j*_, although each of the *s*_*j*_ is not required to be represented by the same basis.

For fitting purposes, the models in Table 1 can be expressed as GLMs. This amounts to minimizing the penalized deviance function


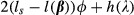
(3)

where *l*_s_ is the likelihood of the saturated model, *l*(***β***) is the likelihood of the model equation 1 with ***β*** also containing parameters related to the nonparametric smooths, *ϕ* is a scale parameter, and *h*(*λ*) is a penalizing function of the smoothing parameter vector *λ* quantifying the “wiggliness” of the smooths. The best way to minimize equation 3 is an active area of research for which recent developments (Wood 2008, 2011), combined with ongoing improvements in generalized linear mixed models software (Bates et al. 2012), facilitate their practical use in analyzing phenological data. Older approaches to fitting mixed GAMs, which are often needed for empirical data analyses, rely on penalized quasi likelihood (PQL) fitting techniques (Breslow and Clayton 1993). PQL does not enable calculation of model maximum likelihoods, useful for multimodel inference, and are additionally known to perform poorly for Bernoulli or count data with near zero means (Wood 2008), not uncommon features of phenological data.

Although fitting nonparametric models is increasingly robust and straightforward, significance testing of terms is comparatively less because of the need to invoke large sample asymptotic approximations in deriving the needed formulae. As with standard linear or GLMs, the equations used for computing model smooth term *P*-values are beyond the scope of the review here, and we refer readers to sections 4.8 and 6.6 in Wood (2006) for a tractable development of the ideas and theory at an introductory level. One particular result we will study here is the following. Let **V**_*j*_ be the variance–covariance matrix for a subset of the fitted parameter estimates 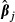 pertaining to a particular smooth 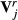 is its pseudoinverse, where *r* is the estimated degrees of freedom of the smooth (see p. 189 and sections 4.8.5 and 6.6 in Wood 2006), then large sample-based theory leads to the distributional result


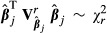
(4)

for obtaining *P*-values on the test that 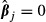; that is, equation 4 provides a means for carrying out a classical significance test for estimating the significance of smooth terms in the model.

A paradigmatic difference emerges in theory leading to the construction of confidence intervals around nonparametric smooths, necessary when characterizing the actual shape of smooth terms *s*_*j*_. Starting with the recognition that the penalization in equation 3 imposes a prior belief constraining the potential “wiggliness” of model equation 1 leads to a Bayesian characterization of description of certainty about the 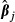, and hence on inference of smooth function of the covariate (see sections 4.8.1–4.8.4 and 6.6 in Wood 2006 and Silverman 1985 for equations and derivations). Let C(α,*t*) be the (1−α)100% Bayesian credible interval of a smooth term *s*(*t*) obtained from asymptotic large sample theory. Then in many situations both theory and simulations (Wahba 1983; Nychka 1988; Wood 2006) indicate that the average coverage probability across the observed data points


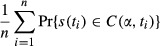
(5)

is often very close to the (frequentist) nominal 1 − α level.

## Simulation Experiments

### Simulation design

We simulated data from which the true underlying functions are known to evaluate how well model term selection procedures based on *P*-values obtained from equation 4, and estimation of smooth functions themselves or exclusion of the zero function using equation 5, might work in practice. Guided by the case study data presented below, we assumed that the presence or absence of a plant structure on individual plants was recorded. The sampling setup assumed 10 years of data collected at regular monthly intervals for 20 individuals without individual heterogeneity. We opted to exclude this heterogeneity to focus on issues related to inference of smooth terms, a universal goal common to applications of nonparametric models, where individual heterogeneity appears to vary considerably from one system/species to another (see the case study results for examples).

**Figure 1:**
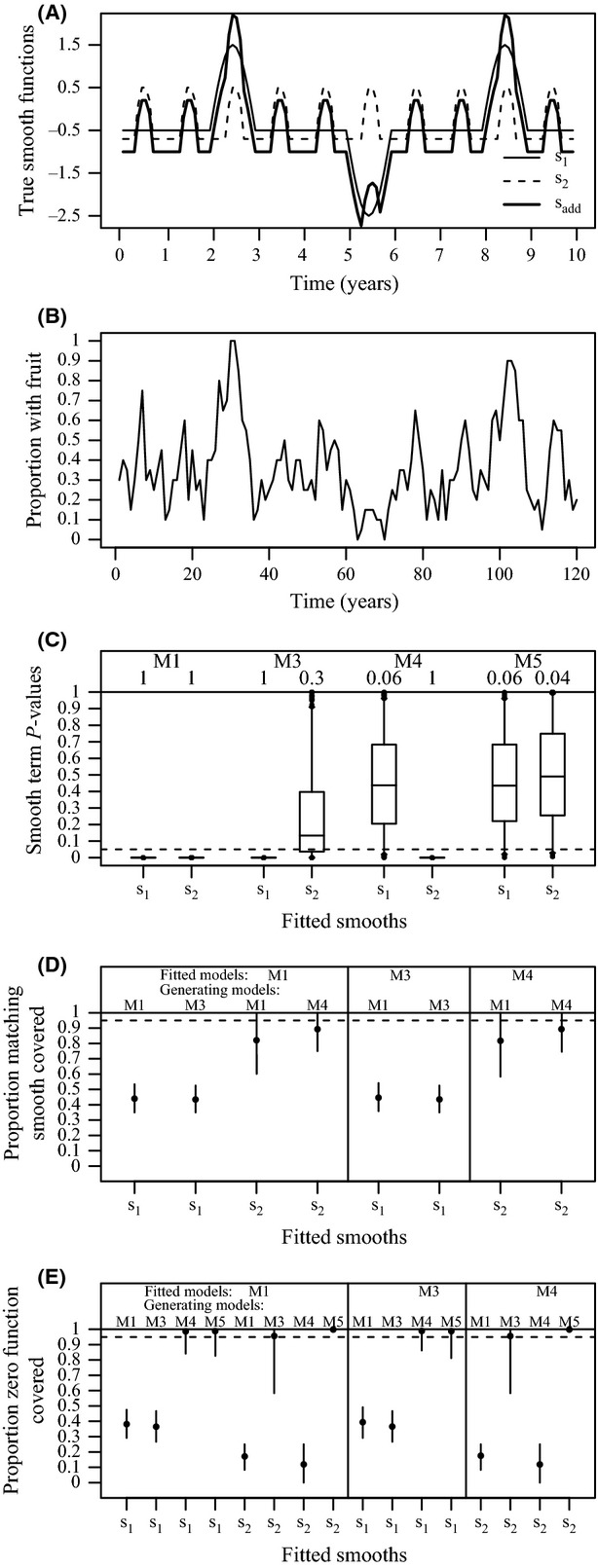
Results from the simulation study. (A) True piecewise smooth functions on the linear scale used in the data simulation with the intraannual cyclic function *s*_2_(*t*) shifted down slightly from its minimum value of −0.5 for clarity. (B) An example time series of the proportion of individuals with fruit in a simulated population (*N*_*s*_ = 20) using the generating model M1. (C) Box plots of *P*-values when testing the significance of smooth terms in a full model M1 fit to synthetic data from the four different data-generating scenarios, listed above each of the corresponding box plots and corresponding to true model structures M1, M3, M4, and M5. Numeric values below the data-generating labels denote the proportion of fitted *P*-values less than 0.05. Box plots are drawn so that the box surrounding the horizontal line, drawn at the median *P*-value estimate, extends across the interquartile range, whiskers extend to the 2.7 and 97.5 percentiles, and points indicate outliers. The horizontal dashed line is drawn at 0.05. (D–E) Points show the average over 1000 simulations of the mean proportion of data points for which the true smooth (panel C) or zero function (panel D) lies within each estimated smooth's 95% credible region at the corresponding data points, across a range of different scenarios of fitted and data-generating models. Vertical lines extend between the 0.0275 and 0.975 quantiles of the 1000 proportions. Note that the different model fitting structures have different possible smooths that are estimated. See Table 1 for model definitions.

Figure 1a shows the functions on a linear scale used to construct data-generating models and include a nonlinear function describing two good years and a bad year relative to a constant probability of fruiting, a periodic function with the same baseline probability of the presence of an ephemeral plant structure, but with regularly occurring and identical peaks intended to mimic seasonal-type emergence of new structures and their additive sum; see the online supplementary material (OSM) in the archives for exact values of these functions. The logistic function was used to obtain expected probabilities from which to generate random data from the additive function or each function separately for each of the 20 plants over the 120 months of observation, and the conical logit link function for *g* was used in model fitting. We also simulated data with constant probability 0.5 (model structure M5) for each plant to show fruit. The model structures used to simulate data are M1 and M3–M5 from which 1000 synthetic data sets were generated each. The simulation study was done in the R programming environment version 2.15.1 (R Development Core Team 2012) and models were fit using the mgcv package (Wood 2006, 2011). Computer code to reproduce this study is available in the OSM.

### Simulation results

Figure 1B shows that model term selection results using *P*-values computed from equation 4 generally provide accurate guidance on the presence of terms in generating data. The primary exception is a tendency to accept intraannual smooth terms more frequently than the nominal expectation of 0.05 when it was not used in the data-generation process (actual proportion of acceptance = 0.18 for model structure M1 fit to model data synthesized from data structure M3); these findings are in line with known deficiencies related to model selection uncertainty (see Discussion on p. 195 in Wood 2006). Thus, although initial model term *P*-values will provide in general accurate inference about which terms to retain, some consideration of each smooth terms confidence intervals is also warranted.

The results on the agreement between asymptotic-based 95% Bayesian credible intervals and actual frequentist-type coverage are shown in Figure 1C–D. Figure 1C shows that the true interannual smooth function *s*_1_(*t*) is not very well estimated, being typically outside of the 95% credible interval of the estimated smooth for more than 50% of the data points. For the cyclic smooth function *s*_2_(*t*), there is a much closer agreement between the actual and nominal coverage values, although at between 80% and 90% this is also typically lower than the theoretical 95%. These results are in line with results and discussion presented by Nychka (1988) who illustrated how increased function estimation bias around “kinks” in the true smoothing function leads to coverage lower than the nominal 1-α rate. The kinks in our periodic function *s*_2_ appear not to cause such severe mismatches in coverage rates, presumably in part because there is more information about the function through the repeated observations on single locations of the function *s*_2_. We suggest that the situation of a cyclic underlying true function with repeated samples at a constant interval most closely matches the study of Wahba (1983) for which the frequency interpretations of Bayesian credible intervals hold quite well.

Figure 1D shows the proportion of points for which the credible intervals contain the zero function, and illustrates that when there is a mismatch between the terms in the fitted and generating models, the fitted smooths contain the zero function on average for more than 95% of data points within their 95% credible interval. Given a match between a nonzero smooth function in the generating model and the fitted model, we can expect the zero function to be contained within the 95% credible interval for about 40% of the data for the interannual smooth *s*_1_(*t*), and for about 15% of the data for the intraannual cyclic smooth *s*_2_(*t*).

To summarize, the simulation results suggest that *P*-values are very reliable for detecting smooth functions, but that estimating these functions can be problematic, especially for any potential nonlinear interannual smooths. However, if the smooth term does not exist in the data-generating model, but does in the data-fitting model, the data-fitting model's smooth estimate will contain the zero function in its 95% credible interval for approximately the nominal proportion of observations, so further investigation of smooth functions in relation to the zero function can help detect false acceptance of smooth terms.

## Empirical Studies from an Afromontane Tropical Rainforest

### Data and models

Here, we illustrate the use of nonparametric methods, and GAMMs in particular, to disentangle time-localized perturbations, long-term trends, and seasonality from empirical fruit presence–absence data. Phenology data comes from the fruiting status of individual trees collected monthly within BINP. The study site is located at approximately 01°02′ 46′′S and 29°46′ 20′′E near the Institute of Tropical Forest Conservation at an elevation between 2100 and 2500 meters and is the continuation of a monitoring study initiated in 2005 (Ganas et al. 2009) in which repeated monthly observations of fruit presence–absence status of marked individuals were made along ∼13 km of forest access trails during the 95 months spanning September 2004 to June 2012. For the forest community-level analysis, we used 249 plants that survived throughout the entire study period from 33 species, irrespective of species-level sample size *N*_*s*_. To estimate species population-level statistics, we focused on a subset of focal species for which *N*_*s*_ was at least five which resulted in 229 individuals from 25 species. Table S1 in the OSM summarizes species names and sample sizes. Rainfall and temperature data were collected over a time period prior to the phenenology data collection at the Institute of Tropical Forest Conservation.

**Figure 2:**
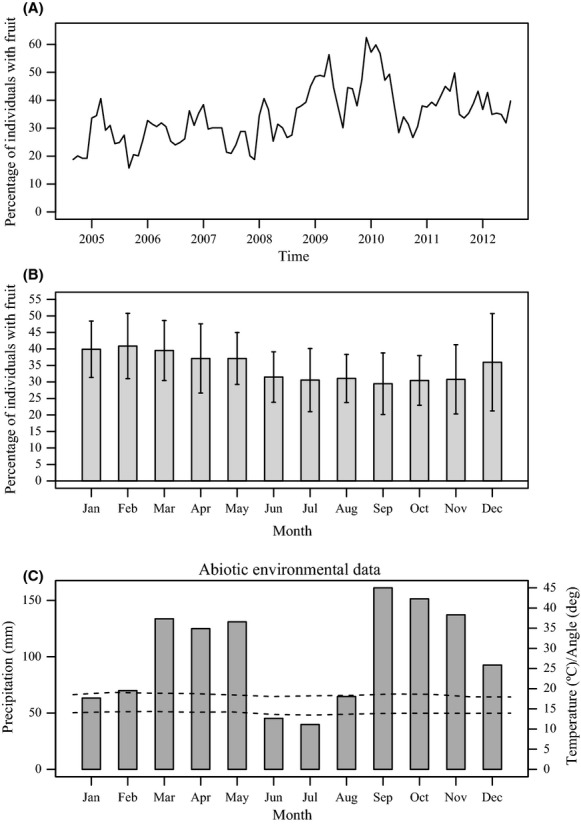
Forest community data. (A) Time-series plot of the proportion of individuals showing fruit for the forest community. (B) Bar plots of the monthly mean proportion of individuals with fruit, with lines extending ±1 SD. (C) Mean monthly rainfall (bars), temperature (dashed lines), and degrees from the zenith of the sun at noon (solid line). Precipitation use data collected from January 1987 through July 2006. Temperature plots use data collected from March 1987 through April 2006.

Figure 2 summarizes the proportion of individual plants with fruits present from tree species of BINP (described in more detail below). There appears to be an upward trend in the percentage of individuals with fruit over time that may or may not be linear (Fig. 2A). A somewhat weak intraannual seasonal pattern is also apparent (Fig. 2B), with peak fruiting occurring just prior to one of the two rainy season (Fig. 2C). Two questions we might ask are whether the seasonality is significant, and, if so, whether the large spikes shown around 2009 and 2010 are associated with the seasonality or are distinct?

Six percent of all possible entries were missing, with a median of four missing data points per plant. We first interpolated these missing data with the smoothed estimates of hidden Markov models (Rabiner 1989) fit to each individual plant separately. We implemented the hidden Markov models and obtained interpolations using the mhsmm package (O'Connell and Højsgaard 2011) for the R version 2.15.1 programming environment (R Development Core Team 2012) using binomial latent and observation state models. This step facilitates use of the entire dataset when incorporating a time-lag dependency in the probability model for individuals showing fruit.

For the empirical data analyses, the models of Table 1 were expanded to accommodate the repeated measurements and include a lag term for temporal autocorrelation. Thus the full model is M1 of Table 1 is expressed as


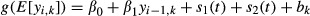
(6)

where the temporal autocorrelation is captured by the *β*_1_*y*_*i*−1*,k*_ term and the unobserved individual random effects *b*_*k*_ for plant *k* are normally distributed with mean zero and standard deviation *σ* with similar modifications to the remaining models. The other models of Table 1 were expanded identically. We viewed temporal lag and random-effects components as control variables and retain our interest in inference about the inter- and intraannual smooths.

Two steps were used to arrive at a final model for the forest aggregated data and each of the species-level analyses. First, a likelihood ratio test (LRT) was used to test the full model (M1) against a null model (M5). Given the acceptance of the full model M1, this model was checked against the remaining models M2–M4 to identify a first model. Second, we further evaluated the significance of smooth terms of the initially selected model based on the extent to which their 95% confidence intervals contained the zero function. Guided by the simulation experiment we might expect that if approximately 10–12 or 2–3 months of the *s*_1_(*t*) and *s*_2_(*t*) functions exclude the zero function, respectively, then these functions are likely to be correctly capturing nonlinear or periodic changes in fruiting patterns.

Models were fit using the gamm4 function from the gamm4 version 0.1-6 (Wood 2012) package for the R version 2.15.1 programming environment (R Development Core Team 2012). Maximum likelihood smooth parameter estimation and a maximum basis dimension of 10 and 8 for the *s*_1_(*t*) and *s*_2_(*t*) smooths, respectively, were chosen as further required choices in model implementation; unreported analyses exploring the larger and smaller choices in these maximal basis dimensions settings revealed no substantive differences, as they should not. The gamm4 function makes direct use of the lmer or glmer functions in the lme4 version 0.999999-0 package (Bates et al. 2012). R code is available from the lead author upon request.

### Empirical results

**Figure 3:**
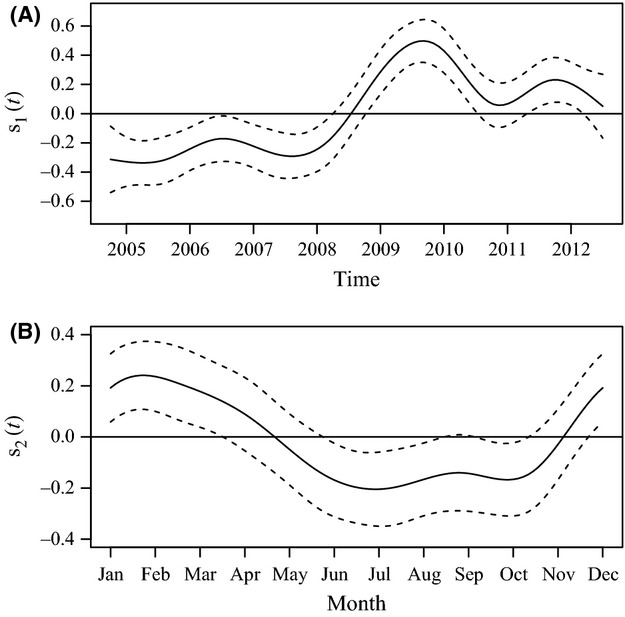
Smooth functions (solid lines) with 95% credible region delineated by the dashed lines estimated from the forest aggregated data for the (A) interannual smooth *s*_1_(*t*) and the (B) intraannual smooth function *s*_2_(*t*), both plotted on the linear scale.

The model inference procedure applied to the forest-level data shown in Figure 2A clearly selected the most complex model, unambiguously rejecting both the null model (*χ*^2^ = 286.67, df = 3, *P* < 0.01) and the linear trend model (*χ*^2^ = 86.12, df = 1, *P* < 0.01). Examination of the *P*-values of the individual smooth terms (*s*_1_(*t*) smooth *χ*^2^ = 235.28, df = 7.92, *P* < 0.01; *s*_2_(*t*) smooth *χ*^2^ = 90.88, df = 4.56, *P* < 0.01) and their confidence intervals (Fig. 3) provided further support for both a nonlinear interannual trend and seasonality. The interannual smooth *s*_1_ showed a positive increase in the probability of fruiting presence across the data set with the most noticeable increase between mid-2008 until 2010 (Fig. 3A), whereas the intraannual smooth *s*_2_ indicated significant seasonality with fruit presence probabilities peaking around January and February and being minimal during July (Fig. 3B), both matching the patterns observed in Figure 2 well by eye.

**Figure 4:**
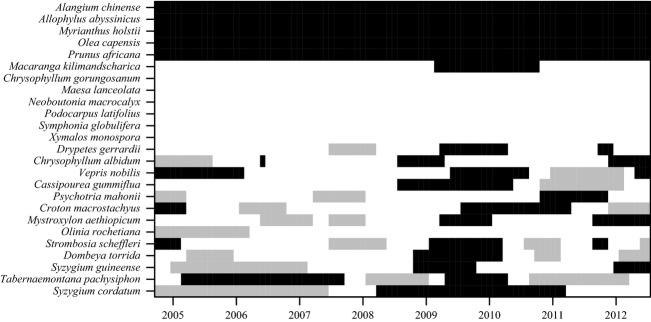
Qualitative directions of the fruiting probability trend based on the interannual term of the final model for each species. For each species and each month, white indicates no trend, black indicates a positive trend, and gray indicates a negative trend. Early 2009 until early 2010 show the greatest amount of positive trending across species, in line with the forest-wide aggregate time-series plot (Fig. 2A) and analysis (Fig. 3A). Species are arranged from those with the most positive trends at the top to the most negative trends at the bottom.

Parallel analyses applied to each of the focal species individually showed a fairly balanced distribution of best model types (Table 2 and Fig. S1 in the OSM). Based on the final model selected, we extracted the time-localized interannual trend information as follows: For species with a significant interannual nonlinear trend (model types M1 and M3), trending was indicated as positive at time *t* if the lower confidence interval of the interannual smooth was greater than zero at time *t*, negative if the upper confidence interval at time *t* was less than zero at time *t*, and not trending otherwise; for species with a significant linear trend (model type M2), the trend was either positive or negative for all *t* depending on the slope coefficient being positive or negative, respectfully; for model types without trending (M4 or M5), the trend at time *t* was defined as not significant for all *t*. Figure 4 summarizes these results (see also Fig. S1 in the OSM) and indicates a cluster in time across species of upwardly trending probabilities from early 2009 until early 2010.

**Table 2:** Selected model counts after applying the GAMM inferential procedure to each of the 25 species with *N*_*s*_ ≥ 5

Models	Based on a likelihood ratio test of model M1 against other models	Further inspection of remaining smooth term confidence intervals
M1	7	6
M2	5	4
M3	9	9*
M4	1	1
M5	3	5

Figure S1 of the OSM provides the detailed graphical and analytical results. See Table 1 for description of models.

* One selected model type M3 had an interannual smooth term converging to a straight line, suggesting a linear upward trend as the best model.

## Discussion

This study first provided a simulation experiment on the utility of using nonparametric methods in a null hypothesis testing framework to study phenological patterns typical of tropical systems, and second, applied GAMMs for an analysis of empirical data. The nonparametric framework proves to be useful because of species with both linear and nonlinear long-term trends, diverse interannual and intraannual seasonal patterns, and nonzero individual random-effects variance.

For the empirical data presented here, the answer to the question posed earlier, is the forest producing more fruits over the course of the study, is yes, but with nontrivial qualifiers. The nonlinearity and seasonality at the forest level, and the many kinds of patterns at the species level make this answer dependent on the plant species. Overall, our study reveals an interannual upward trend in the percentage of individuals with fruits, the qualitative direction of which agrees with a recently detected trend in a nearby lowland forest studied by Chapman et al. (2005). However, the upward trend found here is better understood here as nonlinear mean-level shift with increasing amplitude in the intraannual oscillations, distinct from any regular seasonality. In particular, the years 2009 and 2010 (Fig. 4) fruit production appeared to have been especially high for the most species considered here, and stimulate inquiry into what atypical events relevant for fruit production occurred shortly prior or during this time.

As such, these analyses provide examples of first steps to classifying the winning and losing plant species in response to climate change or other shifting ecosystem properties. At Bwindi, the dry season of 2009 (July–August) was especially dry and hot (M. Robbins, pers. obs.); whether this climatic extreme triggered a response by the plants or pollinators that was beneficial for fruit production remains to be studied. Furthermore, although it is tempting to ascribe the late dry season peak fruiting times associated with the regular seasonality to a lack of light limitation (Wright and van Schaik 1994), it is not automatically the case that the dry season corresponds to increased light availability, and further measurements are needed to resolve whether the seasonality shown here is due to light or water constraints. However, given that the rainy season period is approximately semiannual (Fig. 2C) while regular fruiting fluctuations occur with annual periodicity (Figs. 2B and 3B), it is unlikely that fruiting presence is tied to rainfall in a simple way.

The fruiting pattern complexity observed here implies a complexity relevant for classical topics in ecology and evolution. For instance, while both theoretical (Boyce and Daley 1980; Henson and Cushing 1997; Holt 2008) and experimental (Jillson 1980; Friman and Laakso 2011) studies about population dynamics in regularly pulsing resource environments have provided invaluable insight into underlying mechanisms of population regulation and evolution, comparatively less work has been done for more exotic situations such as those illustrated here. For example, the dynamical consequences for populations dependent on irregular and relatively extreme pulses in resources laid over regular pulses remain generally understudied (but see Holt 2008 for some theoretical forays along these lines).

The models in Table 1 can be expanded to easily include covariate data. However, identifying the mechanistic pathway for a particular covariate to guide model formulation can be challenging. In tropical systems, rainfall and light availability are both leading candidates for resource limitations impacting fruiting (van Schaik et al. 1993; Wright and van Schaik 1994). Given these data, which are rarely available in African tropical research projects, but hopefully will be as automated weather recording stations become increasingly practical (e.g., see the Tropical Ecology Assessment and Monitoring Network program at http://www.teamnetwork.org/), it would be straightforward to incorporate such covariate information into the dependencies of the smooth functions in the models of Table 1. We note that from a modeling perspective these two covariates may be smooth functions of each other because seasonality in light and rainfall is often related, which in the past has been problematic. Given the increasingly robust numerical procedures for fitting nonparametric regression models (Wood 2008, 2011), such problems of concurvity should be relatively minimal. We also note that GAMM fitting technology allows modeling interactions among fixed-effect predictors in all the standard ways ecologists are familiar with from GLM-type analyses, so that in principle the complexity of allowed models under this framework is limited only by the available data and the appropriateness of a hypothesis.

Phenological analyses using GAMMs rely on the theoretical and computational advancements of inference for both additive mixed (Wood 2006, 2011) and generalized linear mixed (Bates et al. 2012) models which makes fitting syntax intuitive and optimization rapid. In some cases such as count data of individual fruits, data would be better modeled by Poisson distributions. The software used here also accommodates Poisson, quasi Poisson, and negative binomial distributions, all useful for cases where data are counts and potentially overdispersed. Zero-inflated nonparametric models (Liu and Chan 2010) and GAMs for location, scale, and shape (Rigby and Stasinopoulos 2005) offer options for modeling such data nonparametrically (e.g., Hudson et al. 2011), although examples of such models with periodic smooths and random effects have yet to be provided.

## Supporting Information

**Figure S1.** Shows species-level data plots and model M1 smooth terms. R code is provided to reproduce the simulation study and empirical analyses at the species level.

**Table S1.** Lists species names and sample sizes for the forest community data.
