# Supplementary material for: Generalized additive mixed models for disentangling long-term trends, local anomalies, and seasonality in fruit tree phenology
Source: Ecol Evol. 2013 Aug 2;3(9):3141–51. doi: 10.1002/ece3.707 (PMC3790557; doi:10.1002/ece3.707)

Figure S1

Species: *Alangium chinense*
$N_S = 7$
Initial model = M2
Selected model = M2

Species: *Allophylus abyssinicus*
$N_S = 7$
Initial model = M2
Selected model = M2

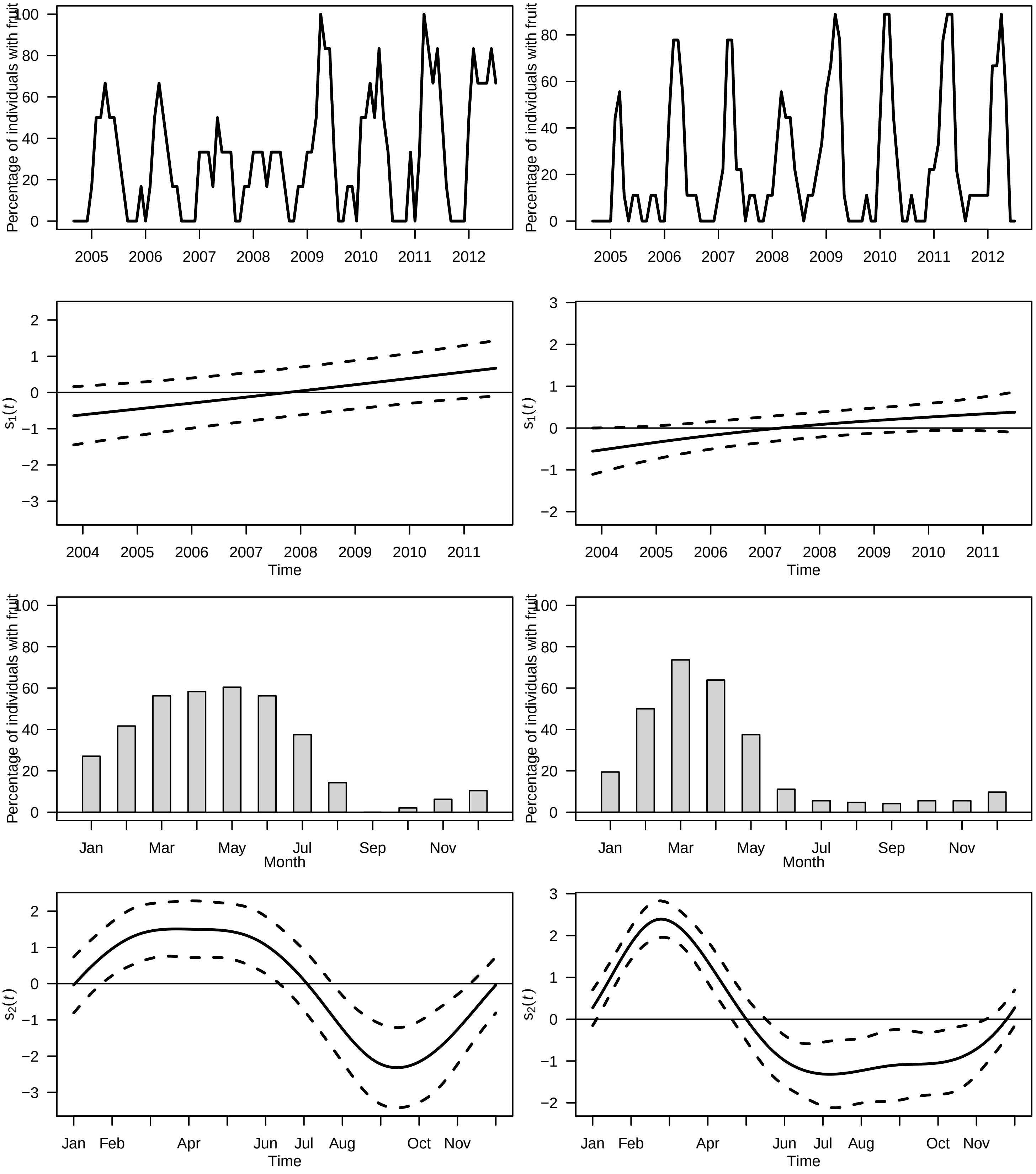

Figure S1

Species: *Cassipourea gummiflua*
$N_S = 7$
Initial model = M1
Selected model = M1

Species: *Chrysophyllum albidum*
$N_S = 7$
Initial model = M1
Selected model = M1

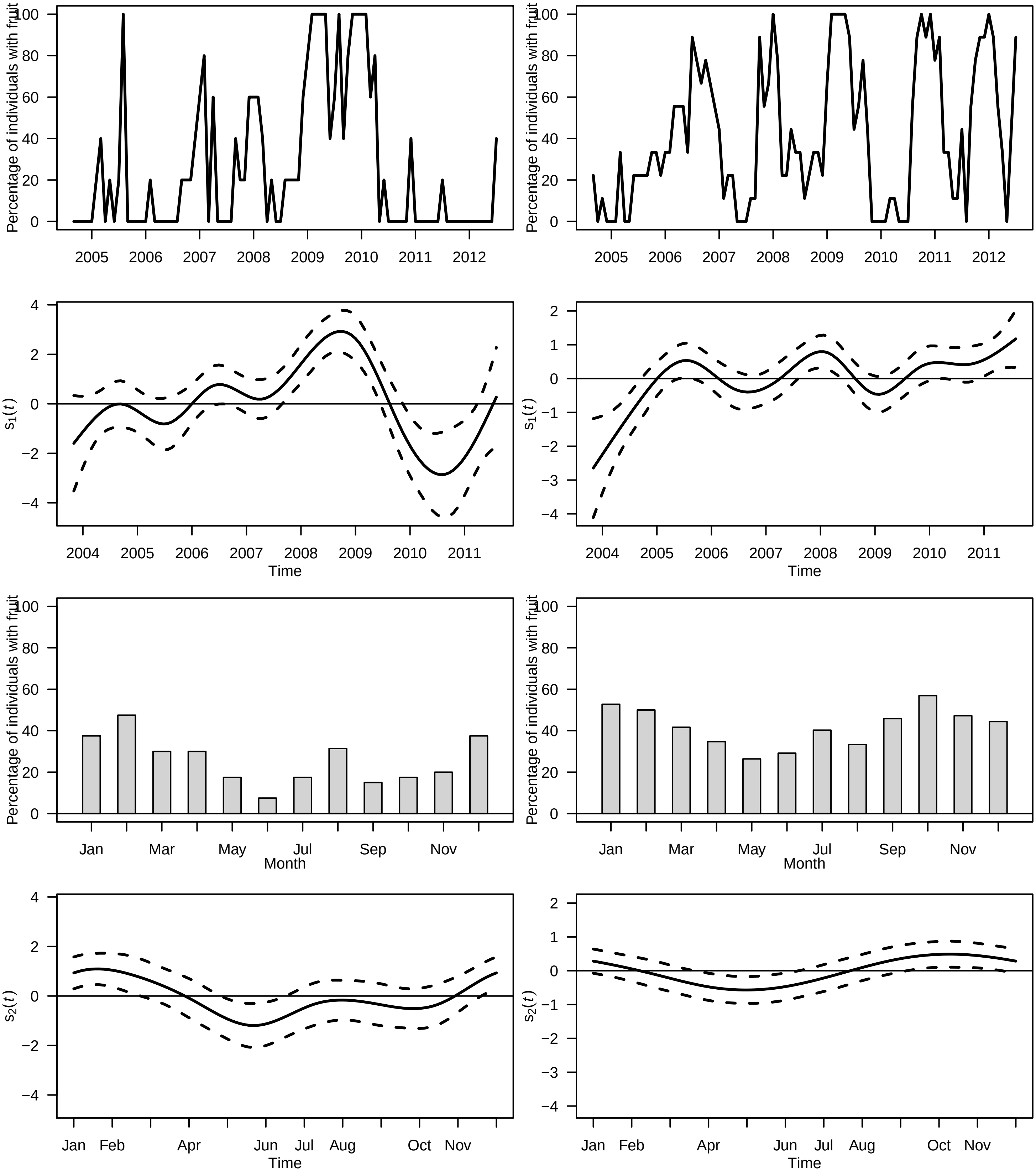

Figure S1

Species: *Chrysophyllum gorungosanum*
$N_S = 7$
Initial model = M5
Selected model = M5

Species: *Croton macrostachyus*
$N_S = 7$
Initial model = M1
Selected model = M1

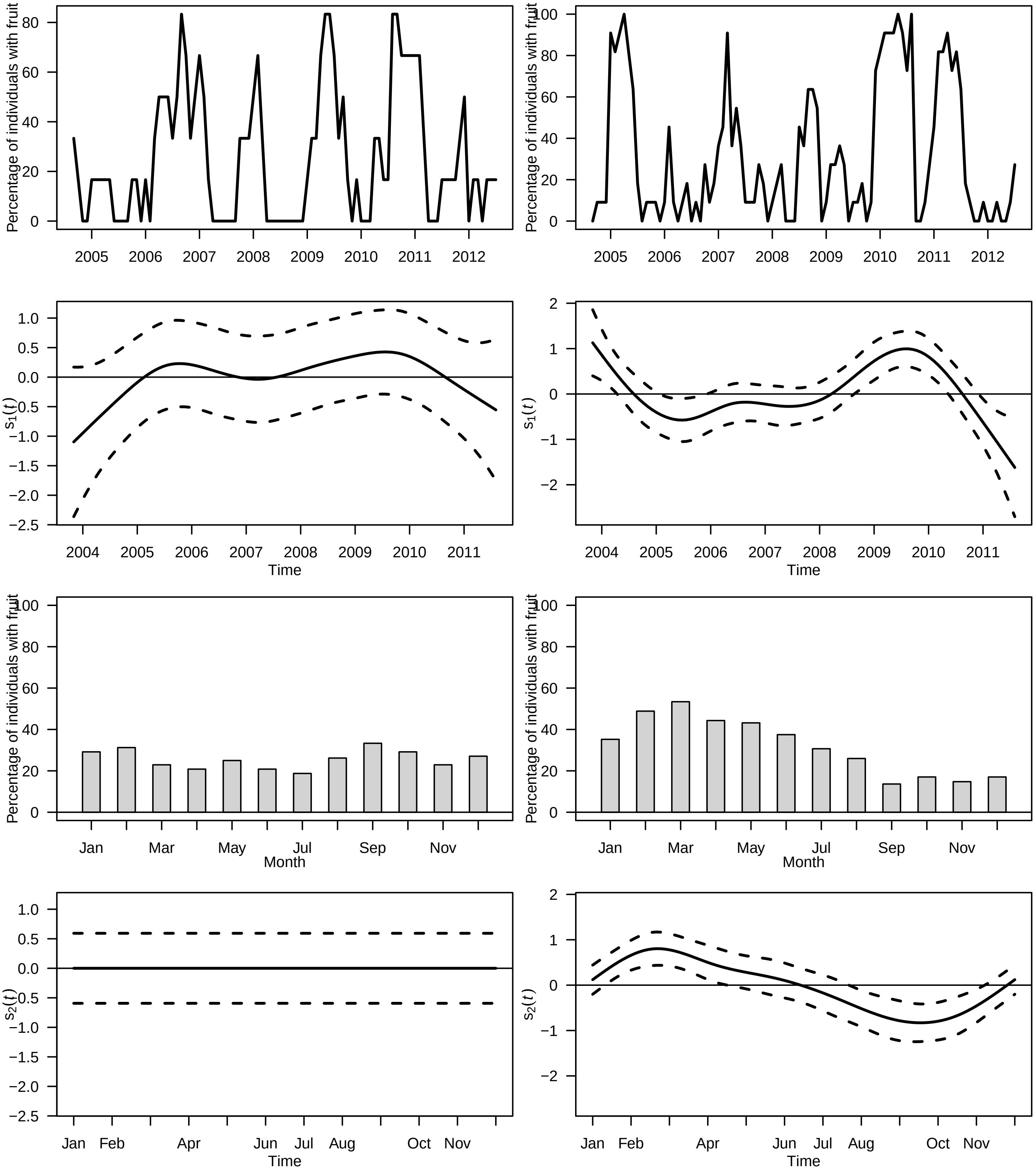

Figure S1

Species: *Dombeya torrida*
$N_S = 7$
Initial model = M3
Selected model = M3

Species: *Drypetes gerrardii*
$N_S = 7$
Initial model = M3
Selected model = M3

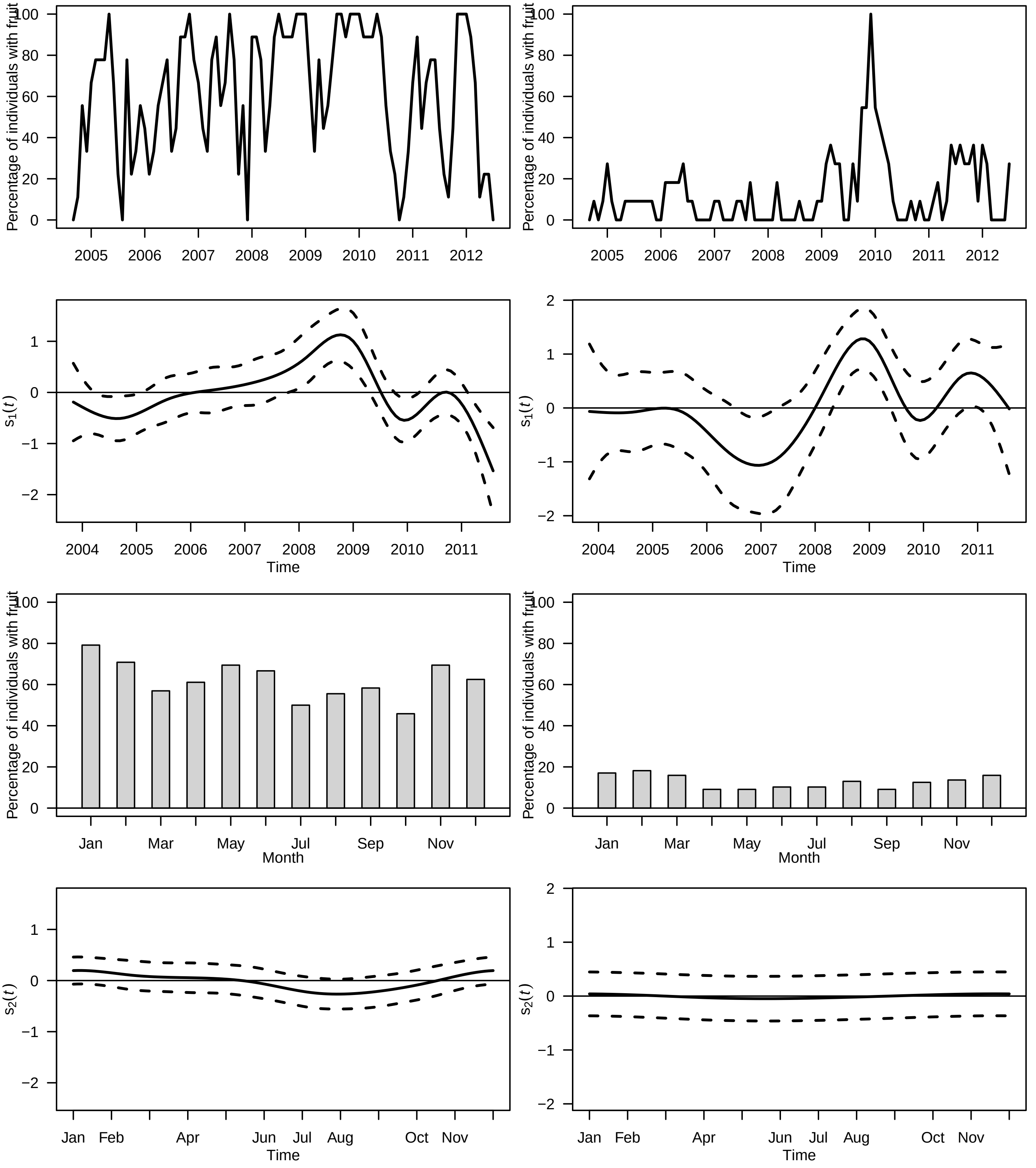

Figure S1

Species: *Macaranga kilimandscharica*
$N_S = 7$
Initial model = M3
Selected model = M3

Species: *Maesa lanceolata*
$N_S = 7$
Initial model = M5
Selected model = M5

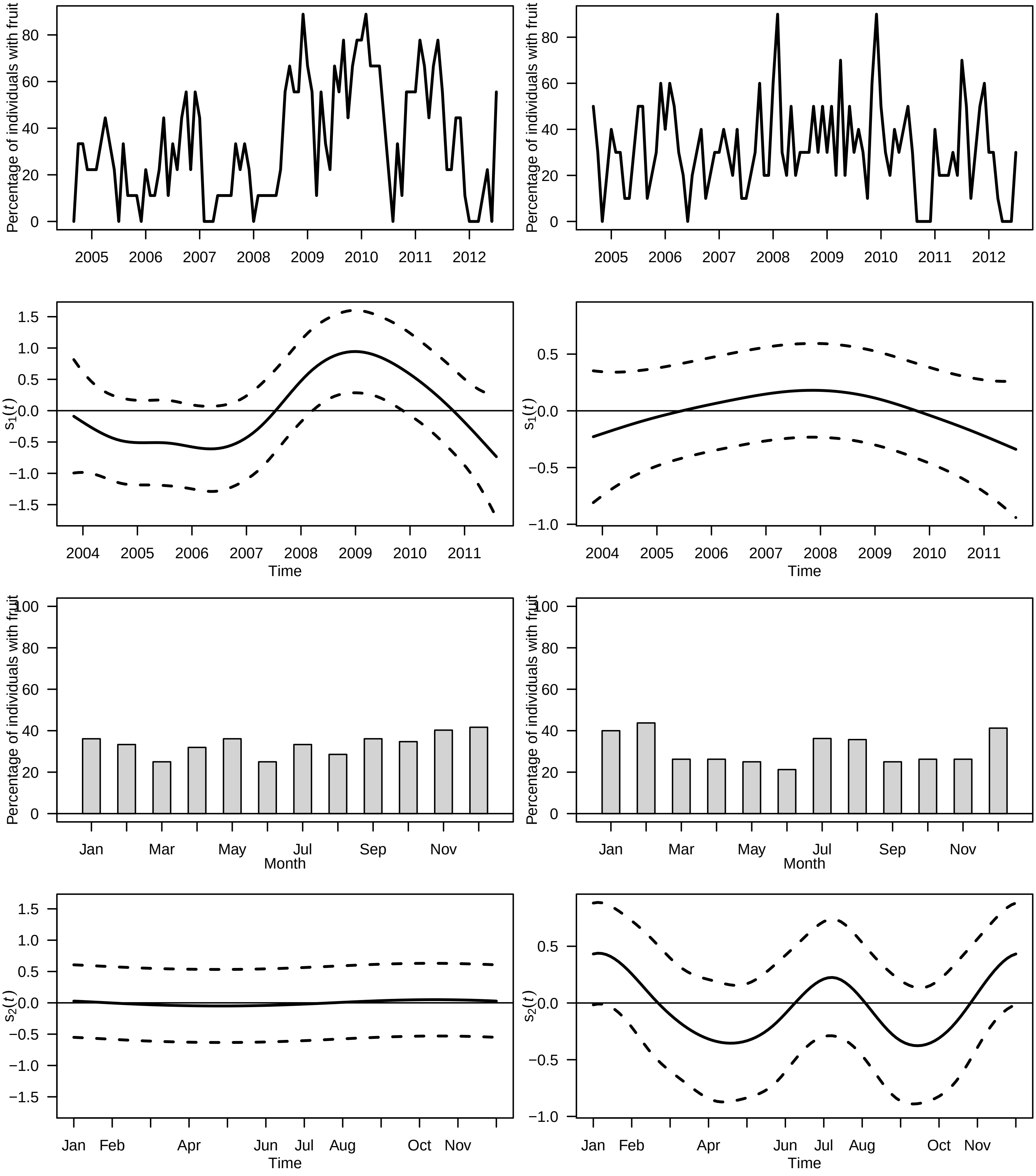

Figure S1

Species: *Myrianthus holstii*
$N_S = 7$
Initial model = M2
Selected model = T+M5

Species: *Mystroxylon aethiopicum*
$N_S = 7$
Initial model = M1
Selected model = M3

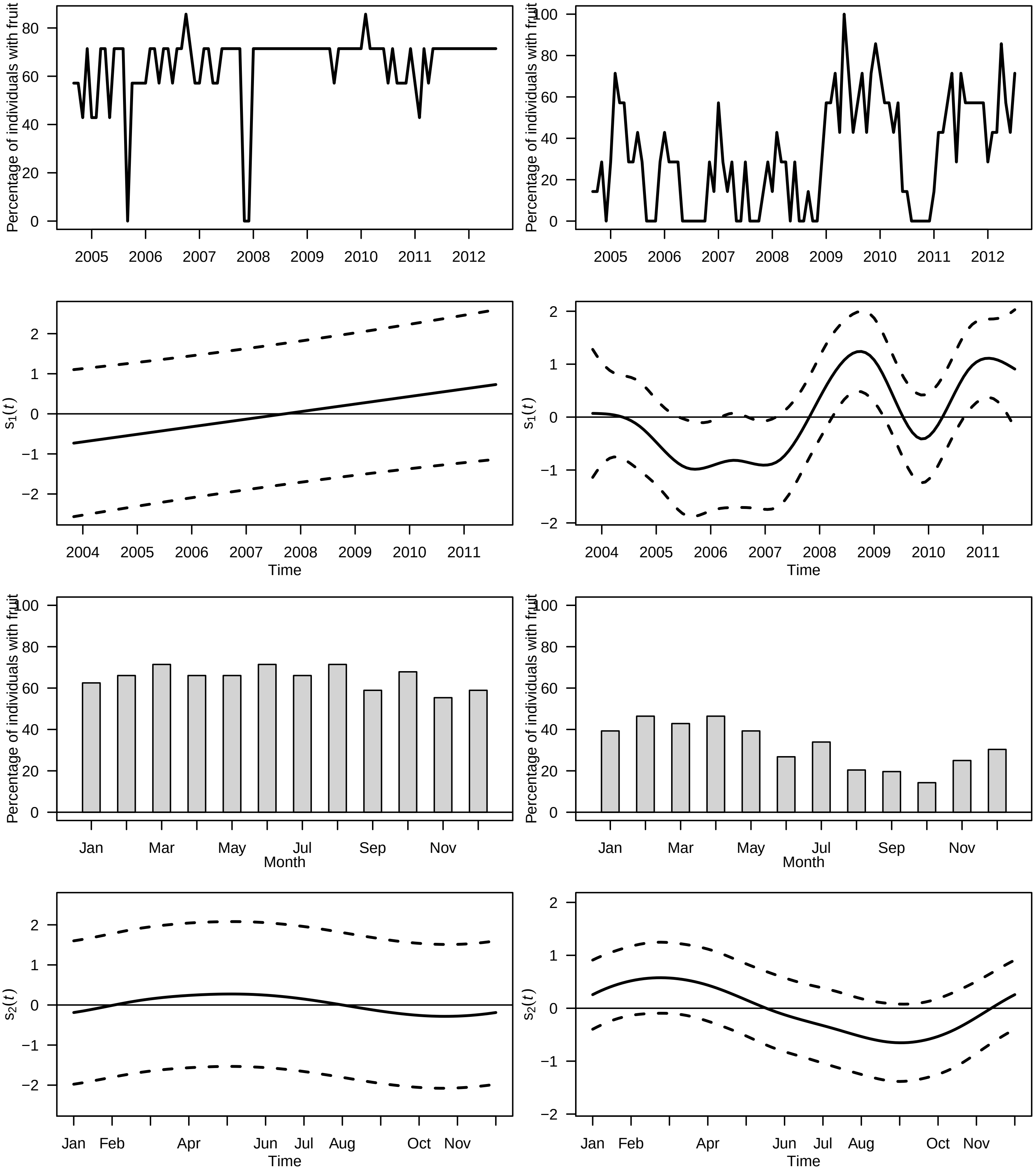

Figure S1

Species: *Neoboutonia macrocalyx*
$N_S = 7$
Initial model = M4
Selected model = M4

Species: *Olea capensis*
$N_S = 7$
Initial model = M2
Selected model = M2

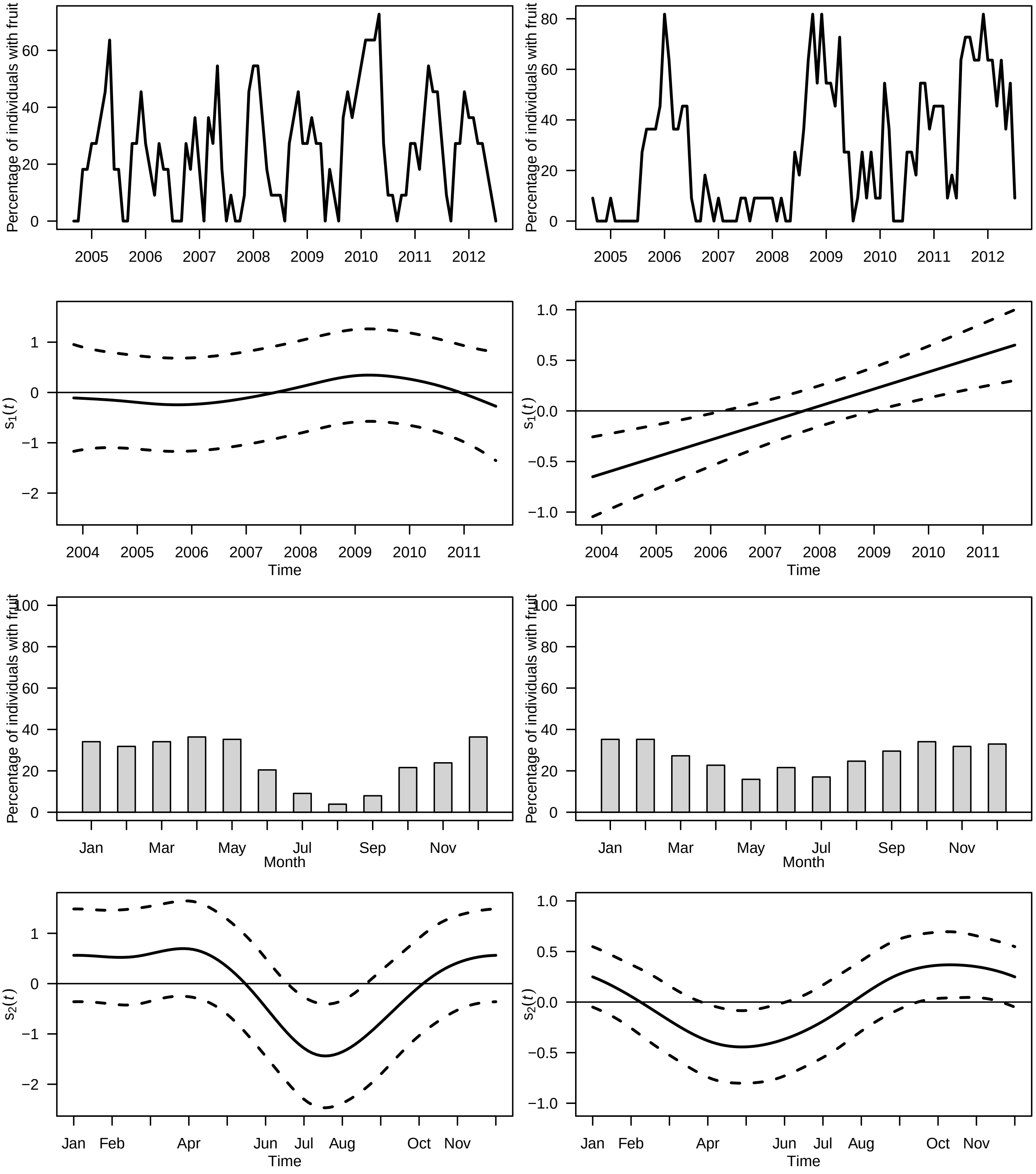

Figure S1

Species: *Olinia rochetiana*
$N_S = 7$
Initial model = M3
Selected model = M3

Species: *Podocarpus latifolius*
$N_S = 7$
Initial model = M3
Selected model = M5

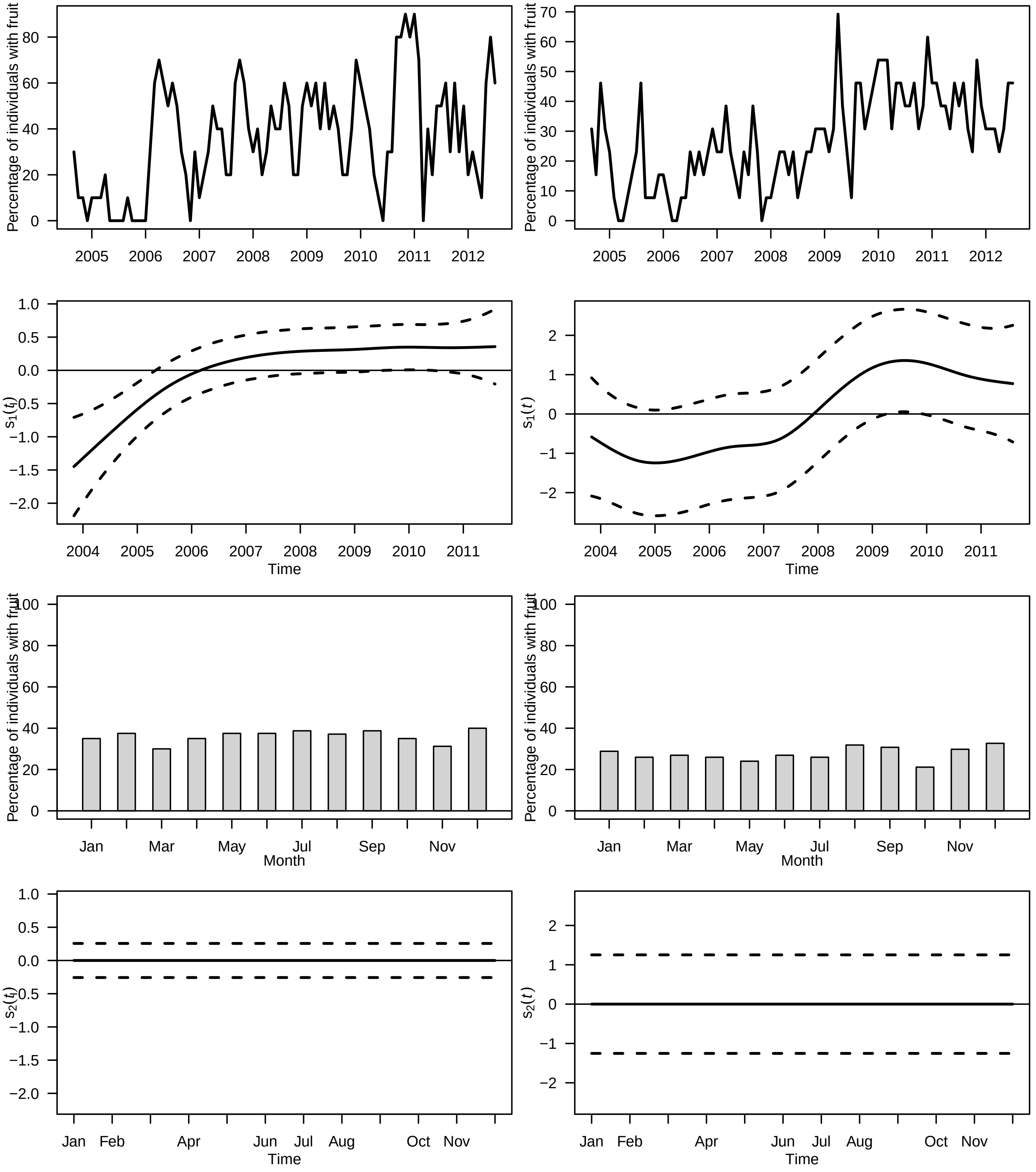

Figure S1

Species: *Prunus africana*
$N_S = 7$
Initial model = M2
Selected model = M2

Species: *Psychotria mahonii*
$N_S = 7$
Initial model = M1
Selected model = M1

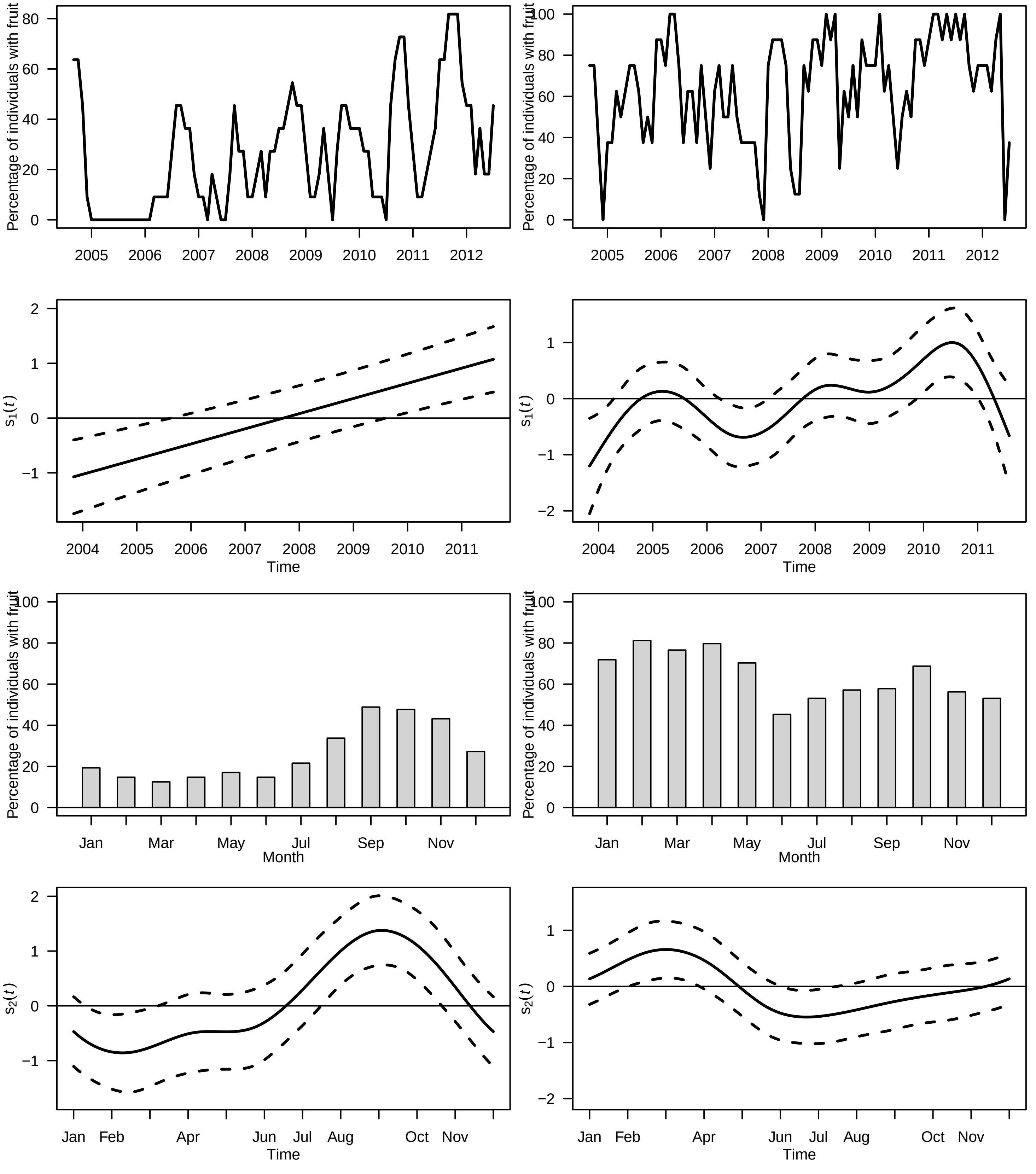

Figure S1

Species: *Strombosia scheffleri*
$N_S = 7$
Initial model = M1
Selected model = M1

Species: *Symphonia globulifera*
$N_S = 7$
Initial model = M3
Selected model = M5

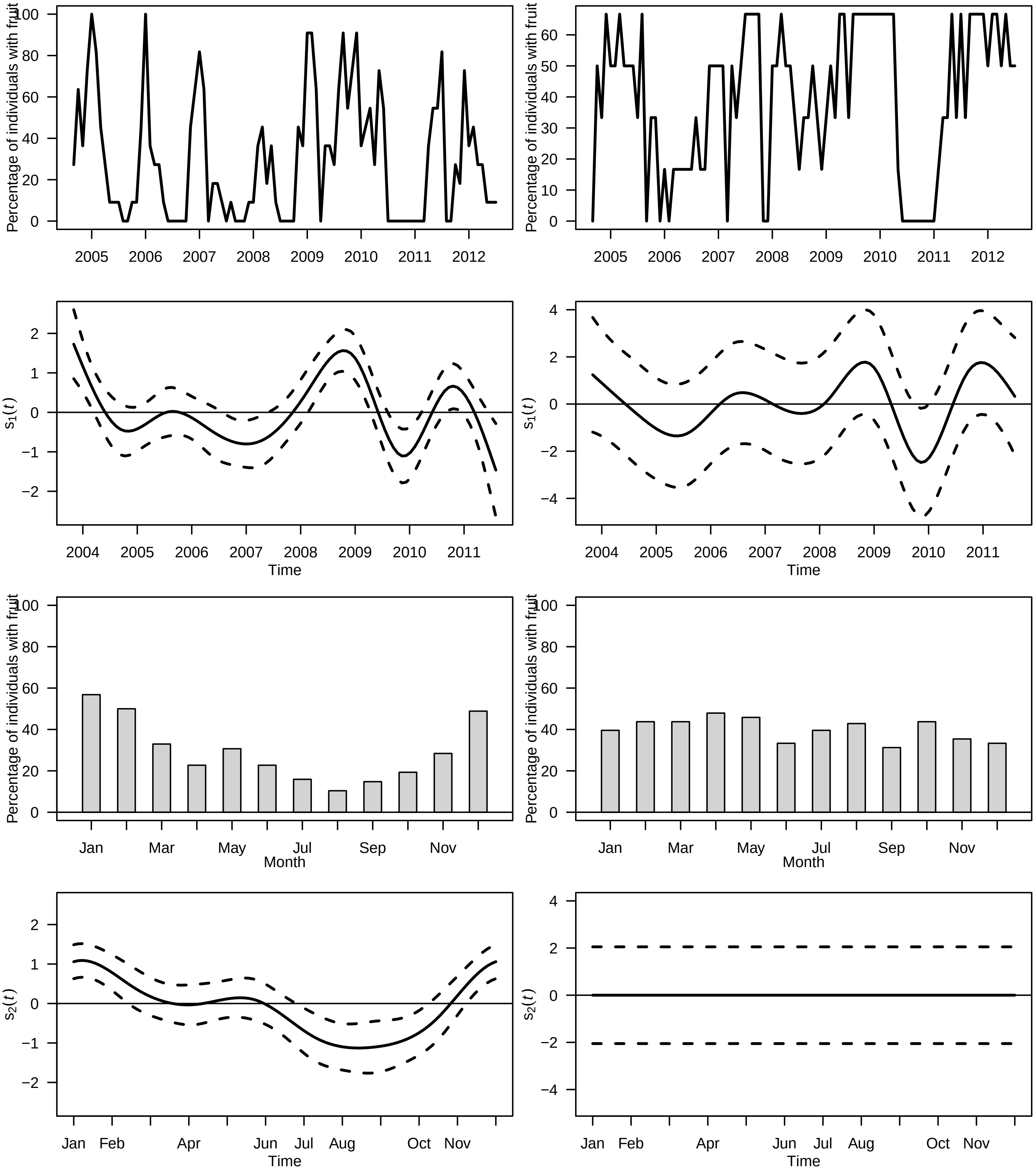

Figure S1

Species: *Syzygium cordatum*
$N_S = 7$
Initial model = M3
Selected model = M3

Species: *Syzygium guineense*
$N_S = 7$
Initial model = M1
Selected model = M1

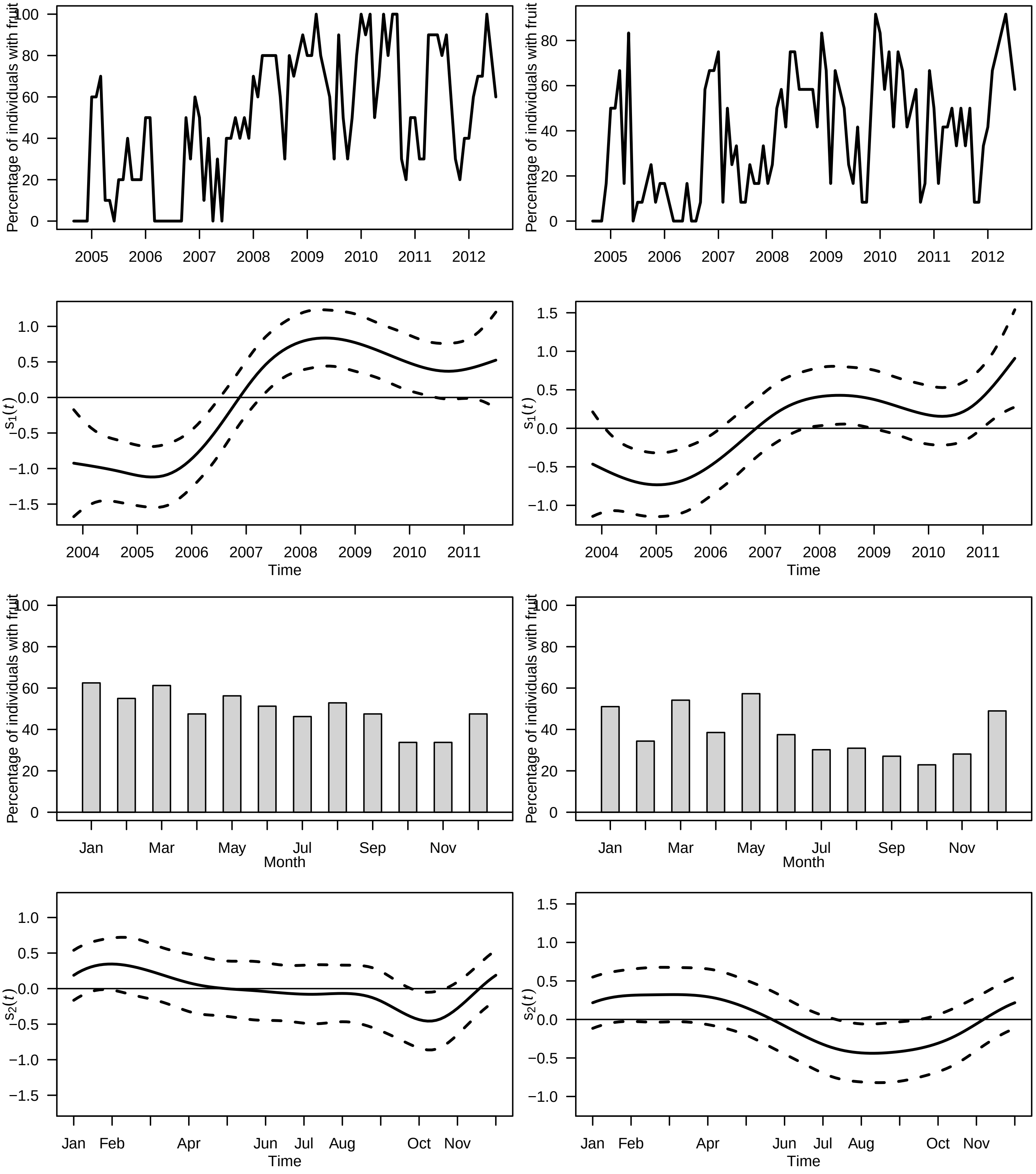

Figure S1

Species: *Tabernaemontana pachysiphon*
$N_S = 7$
Initial model = M3
Selected model = M3

Species: *Vepris nobilis*
$N_S = 7$
Initial model = M3
Selected model = M3

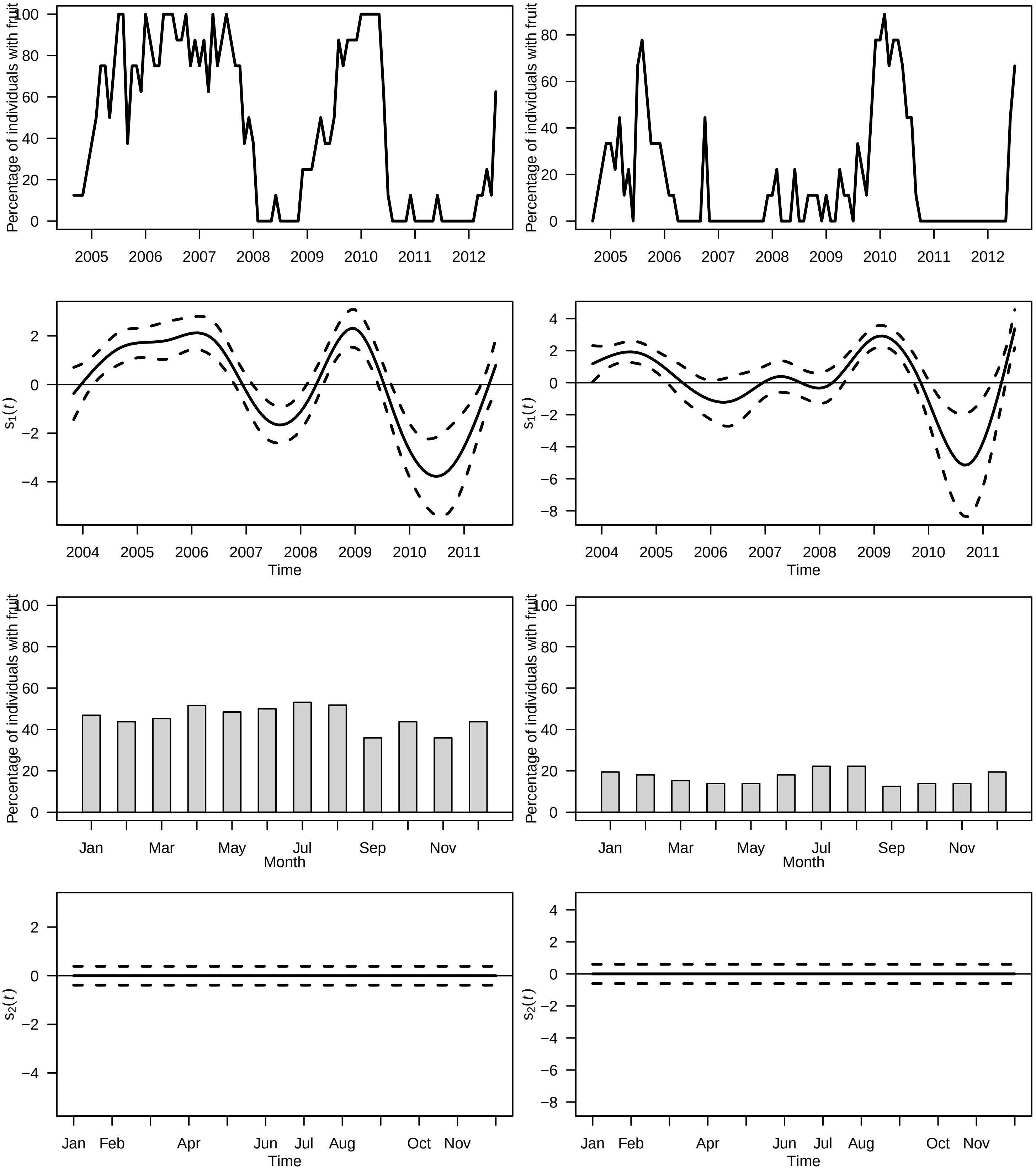

Figure S1

Species: *Xymalos monospora*
$N_S = 7$
Initial model = M5
Selected model = M5

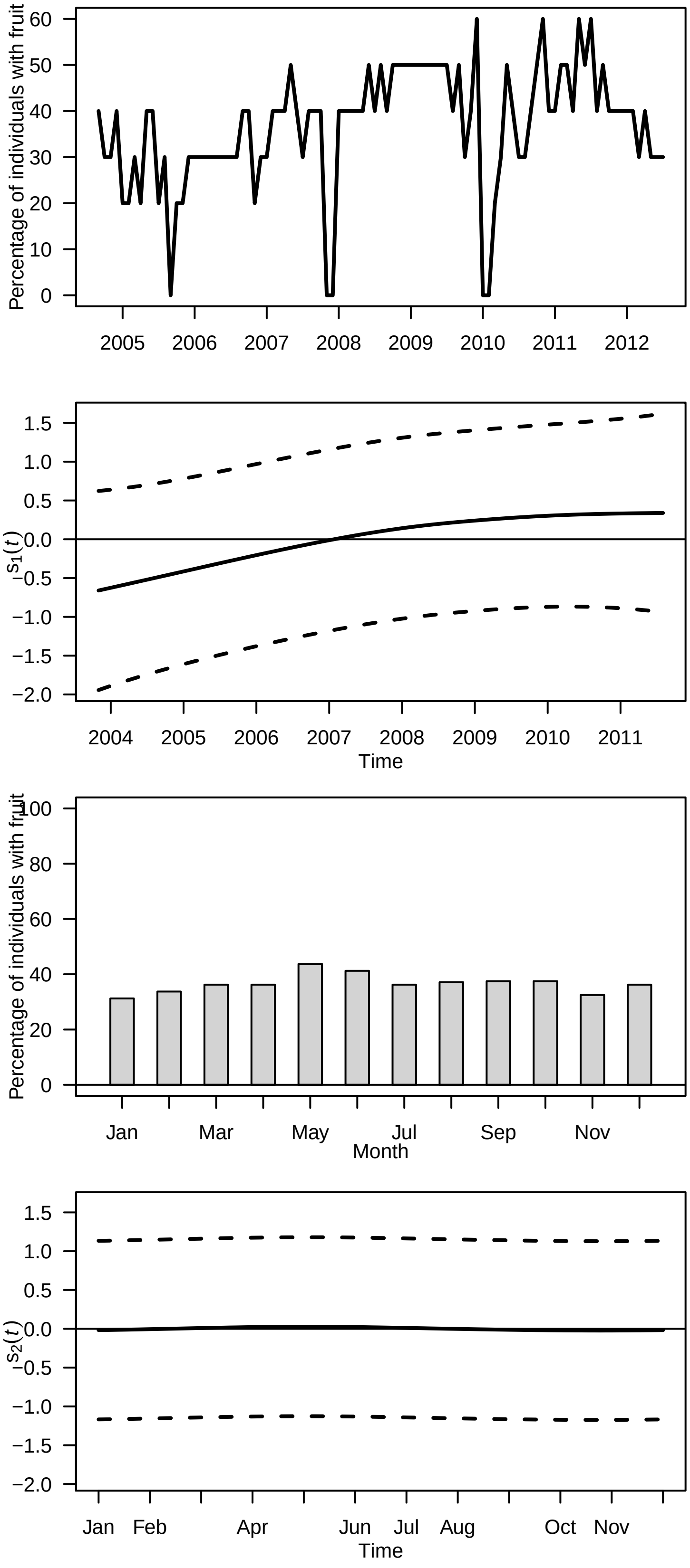

Supplement: Supplementary file 1 [file ece30003-3141-SD1.pdf]
