# Supplementary material for: Generalized additive mixed models for disentangling long-term trends, local anomalies, and seasonality in fruit tree phenology
Source: Ecol Evol. 2013 Aug 2;3(9):3141–51. doi: 10.1002/ece3.707 (PMC3790557; doi:10.1002/ece3.707)
Supplement: Supplementary file 2 [file ece30003-3141-SD2.pdf]

Supplementary material

Generalized additive mixed models for disentangling long term trends, local anomalies, and seasonality in fruit tree phenology

Leo Polansky and Martha M. Robbins

Table S1- Species and sample sizes

Species	N_s
Alangium chinense	6
Allophylus abyssinicus	9
Allophylus ferrugineus	3
Bridelia micrantha	4
Cassipourea gummiflua	5
Chrysophyllum	1
Chrysophyllum albidum	9
Chrysophyllum gorungosanum	6
Croton macrostachyus	11
Dombeya torrida	9
Drypetes gerrardii	11
Ficus	4
Ficus craterostoma	3
Ficus densistipulata	2
Ficus thonningi	1
Harungana madagascariensis	2
Macaranga kilimandscharica	9
Maesa lanceolata	10
Myrianthus holstii	7
Mystroxylon aethiopicum	7
Neoboutonia macrocalyx	11
Olea capensis	11
Olinia rochetiana	10
Podocarpus latifolius	13
Prunus africana	11
Psychotria mahonii	8
Strombosia scheffleri	11
Symphonia globulifera	6
Syzygium cordatum	10
Syzygium guineense	12
Tabernaemontana pachysiphon	8
Vepris nobilis	9
Xymalos monospora	10
